# P-622. Population-Based Longitudinal Maternal and Birth Cohort of Prevalent Respiratory Tract Infections in Children from 0 to 5 years of age

**DOI:** 10.1093/ofid/ofaf695.835

**Published:** 2026-01-11

**Authors:** Eduardo Lopez-Medina, Rodrigo De Antonio, Xavier Saez-Llorens, Pio López, Jose Jimeno, Carlos Gonzalez, Erika Cantor, Osvaldo Reyes, Manuel S Ordoñez, Diana M Dávalos, Colin Kietzman, Marie Wehenkel, Bart G Jones, Flor M Munoz, Octavio Ramilo, Asuncion Mejias

**Affiliations:** Centro de Estudios en Infectología Pediátrica CEIP, Departamento de Pediatría, Universidad del Valle, Clínica Imbanaco, Grupo Quironsalud, Colombia., Cali, Valle del Cauca, Colombia; Cevaxin, Panama City, Panama, Panama; Hospital del Niño Dr. José Renán Esquivel, Panama City, Panama, Panama; Centro de Estudios en Infectolgia Pediatrica CEIP, Cali, Valle del Cauca, Colombia; Vaxtrials part of the Emmes group, Panama City, Panama, Panama; Centro de Estudios en Infectolgia Pediatrica CEIP, Cali, Valle del Cauca, Colombia; Pontificia Universidad Javeriana, Centro de Estudios en Infectolgia Pediatrica CEIP, Cali, Valle del Cauca, Colombia; Hospital Santo Tomás, Panama, Panama, Panama; Universidad del Valle, Cali, Colombia, Popayán, Cauca, Colombia; Centro de Estudios en Infectolgia Pediatrica CEIP, Cali, Valle del Cauca, Colombia; St. Jude, Memphis, Tennessee; St. Jude Children's Research Hospital, Memphis, Tennessee; St. Jude, Memphis, Tennessee; Baylor College of Medicine Houston, Dallas, Texas; St. Jude Children's Research Hospital, Memphis, Tennessee; St Jude Children's Research Hospital, Memphis, TN

## Abstract

**Background:**

In LMIC, respiratory tract infections (RTI) are a leading cause of child morbidity/mortality, yet their epidemiology remains poorly defined. We established a birth cohort to investigate RTI and factors associated with progression to lower RTI (LRTI) at CEIP (Cali, Colombia) and CEVAXIN (Panama City, Panama) study centers; this abstract summarizes initial findings.

**Table 1a).**
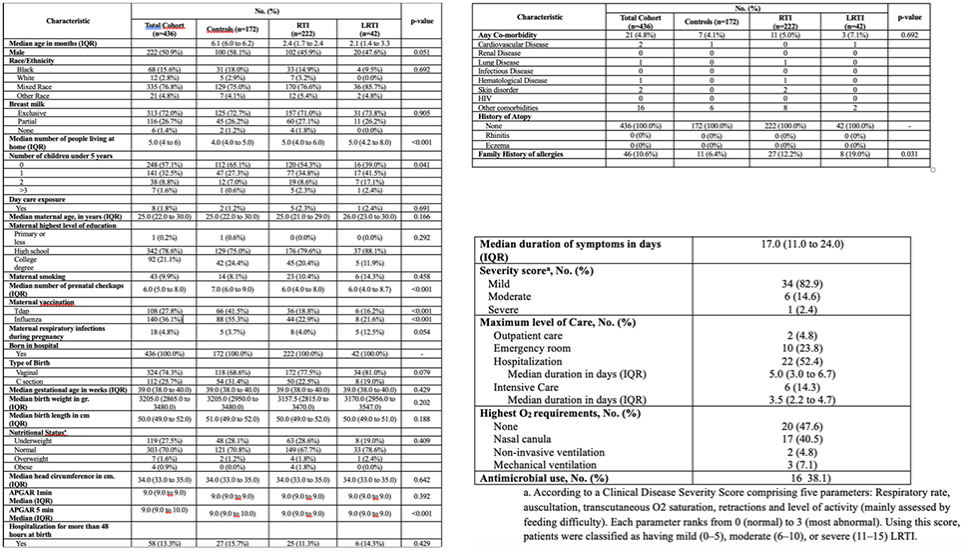
Demographic and clinical characteristics 1b). Outcomes of infants with LRTI

**Figure:**
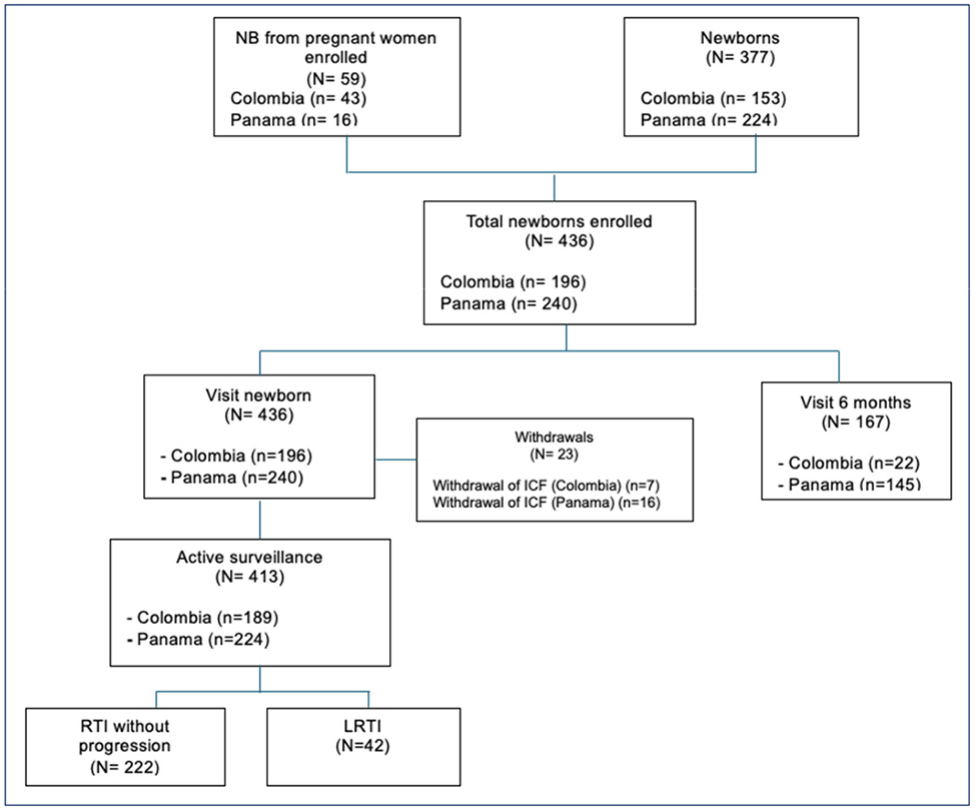

**Methods:**

This ongoing cohort study enrolled and followed pregnant women and newborns from May 2024 to Feb 2025. Infants underwent routine study visits every 6 months and twice-weekly RTI (cough or coryza) and LRTI (+tachypnea/difficulty breathing) surveillance via electronic app. Nasal swabs (NS) collected from symptomatic infants within 4 days of symptom onset, and asymptomatic controls, were analyzed via the TrueMark™ Respiratory Panel 2.0 (OpenArray™), targeting 31 pathogens.

**Figure:**
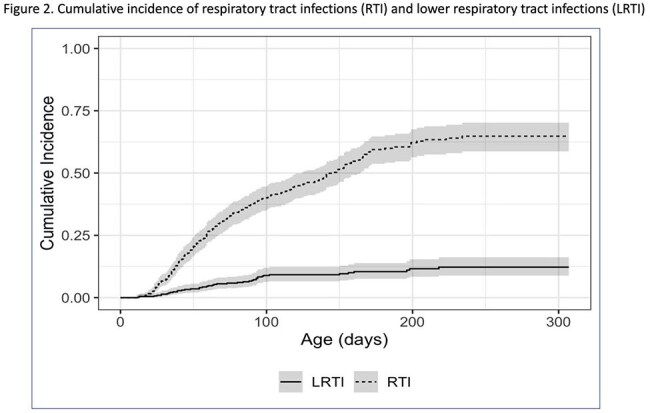

**Figure:**
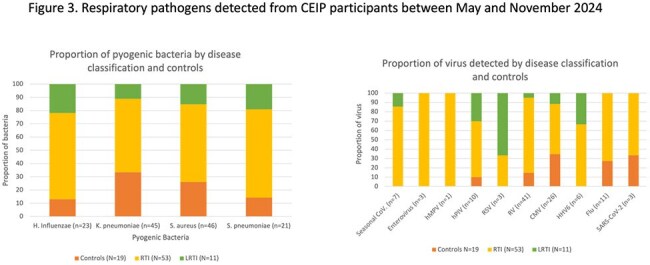

**Results:**

Among 436 infants (59 (13.5%) born to enrolled women (Fig. 1)), RTI occurred in 264 (61%), with 42 (16%) progressing to LRTI. Cumulative incidence of RTI and LRTI by 250 days of life was 65% and 12%, respectively (Fig. 2). LRTI was associated with higher number of household members, fewer prenatal visits, lower maternal vaccination and family history of allergy (Table 1a). Among LRTI cases, 14% required ICU admission, and 38% received antibiotics (Table 1b).

NP were analyzed from 11 LRTI, 53 RTI episodes and 19 controls from CEIP participants followed between May and Nov 2024. Bacteria were identified in NS from 82% of LRTI episodes and 100% of controls. Common viruses were rhinovirus and cytomegalovirus in symptomatic and control infants. RSV was detected at low frequencies, exclusively in symptomatic infants (18% of LRTI, 2% RTI, 0% controls) (Figure 3).

**Conclusion:**

There was a high incidence of RTIs that often progressed to LRTIs; this progression was influenced by prenatal care interventions. Both viruses and bacteria, were identified in NS in cases and controls. The role of NS bacteria in LRTI pathogenesis—especially during co-infections with respiratory viruses—remains a research gap. This study will assess comprehensive clinical and molecular data, including cytokines, antibody profiling, transcriptomics, and immune cell phenotype analyses to enhance understanding of disease activity and risk of RTI progression.

**Disclosures:**

Flor M. Munoz, MD, Merck: Advisor/Consultant|Pfizer: Advisor/Consultant|Pfizer: Grant/Research Support Octavio Ramilo, MD, Merck: Advisor/Consultant|Merck: Grant/Research Support|Merck: Honoraria|Moderna: Advisor/Consultant|Pfizer: Advisor/Consultant|Pfizer: Honoraria|Sanofi: Advisor/Consultant Asuncion Mejias, MD, PhD, MsCS, Enanta: Advisor/Consultant|Merck: Grant/Research Support|Moderna: Advisor/Consultant|Pfizer: Advisor/Consultant|Sanofi-Pasteur: Advisor/Consultant

